# MicroRNAs in Head and Neck Cancer

**DOI:** 10.1155/2013/650218

**Published:** 2013-10-23

**Authors:** Keziah John, Jennifer Wu, Bing-Wei Lee, Camile S. Farah

**Affiliations:** ^1^The University of Queensland, School of Dentistry, Brisbane, QLD 4000, Australia; ^2^The University of Queensland, UQ Centre for Clinical Research, Herston QLD 4029, Australia

## Abstract

microRNAs (miRs) are small noncoding single-stranded RNAs, about 19–25 nucleotides long. They have been shown to be capable of altering mRNA expression; thus some are oncogenic or tumour suppressive in nature and are regulated by cellular and epigenetic factors. The molecular pathogenic pathway of many cancers has been modified since the discovery of miRs. Head and neck squamous cell carcinoma (HNSCC), the sixth most common cancer in the world, has recently been associated with infection by the human papillomavirus (HPV). miR expression profiles are altered in the transition from dysplasia to carcinoma, with some changes being specific to the underlying risk factor. This difference is particularly significant in HPV-positive HNSCC where host miRs are modulated by the virus, creating a different profile to HPV-negative HNSCC. Saliva, as an easily collected proximal biofluid containing numerous miRs, presents an attractive noninvasive diagnostic tool in detecting HNSCC and determining prognosis. Furthermore, miRs may play a role in the analysis of surgical margins for residual tumour extension and in the development of novel miR-based therapeutic targets and agents.

## 1. Introduction

Head and neck cancer (HNC) is the 6th most common cancer in the world [1], referring to cancers of the aerodigestive tract, including lip, oral cavity, nasal cavity, paranasal sinuses, pharynx, larynx, oropharynx, hypopharynx, salivary glands, and local lymph nodes [2]. 90% of all HNCs are squamous cell carcinomas (HNSCCs), arising from the mucosal lining in these regions [2]. 80–90% of these are related to prolonged alcohol and tobacco use, while 30–50% have been associated with the human papillomavirus (HPV) [3], with type 16 being the most common type detected in HNSCC [2]. There is significant geographic variation in its incidence, with South-East Asia, the Pacific regions, Latin America, and parts of Central and Eastern Europe presenting a higher incidence than other regions; for instance, HNSCC is the most common cancer type in India, accounting for 40% of all malignancies [4, 5]. The 5-year survival rate of smoking associated with HNSCC is still 30–50%, with survivors experiencing poor quality of life [3]. Furthermore, HNSCC is usually not detected in the early stages of the disease as they may not display clinical symptoms [3, 6]. Hence methods for early detection and diagnosis of lesions with malignant potential [7–9], measures for prevention, and novel treatment methods are instrumental for improving treatment outcomes and patients' quality of life.

miRs are small, single-stranded RNA molecules that were first discovered in 1993, shown to influence the larval development of the nematode *Caenorhabditis elegans* by regulating translation through an antisense RNA-RNA interaction [6, 10, 11]. In June 2013, the miRBAse database recorded 1600 *Homo sapiens *miRs [12]. Although they are noncoding, they are considered to influence the expression of many protein-coding genes in the human genome [3]. miRs are associated with mRNA translation and degradation, influencing organ development, cell differentiation, proliferation, apoptosis, and stress responses [10, 13–15]. Multiple miRs can target one mRNA, while one miR can influence mRNA transcripts of numerous genes [3], implicating them in tumour development by modulation of cellular levels of specific oncogenes or tumour suppressor genes [10, 16].

miRs are transcribed by RNA polymerase II (RNA Pol II), producing primary miRs (pri-miRs) which are converted to precursor miRs (pre-miRs) by Drosha (an RNAase III endonuclease) and DiGeorge syndrome critical region gene 8 (DGCR8) [10, 17]. pre-miRs (70–100 nt long) are transported to the cytoplasm by exportin 5 and processed by Dicer (an RNAase III enzyme) and TRBP (a Dicer partner) to form double-stranded (ds) RNA, approximately 22 nt long [10, 17]. This dsRNA consists of mature miR and the complementary strand (miR*) [10]. miRs* are usually degraded but may also be functional [10]. Mature miRs can modulate the translation of protein-coding mRNAs by base pairing to partially complementary regions [10]. It recognizes specific sequences, and, using Argonaute 2 (Ago2), TRBP, and RNA-inducing silencing complex (RISC; a multiprotein complex responsible for site-specific cleavage of the target mRNA), it degrades mRNA repressing its translation [10, 17]. miRs have also been found to interfere with RNA binding functions (decoy activity) in a RISC-independent manner [10, 16, 18].

miR expression is regulated at the transcriptional and posttranscriptional levels, by cellular factors (including c-Myc (oncogenic protein inducing oncomir expression), p53 (tumour suppressor protein inducing tumour suppressor miRs), and E2F) or defects in miR biogenesis machinery (Drosha, DGCR8, exportin 5, Dicer, TRBP, and Ago2) [16, 19]. Changes in miR expression in cancer may be due to changes in activation of gene transcription regulators at the promotor [16, 20] (e.g., HIF-1*α* [21] and PKC*α* [22]), epigenetic regulation by altered DNA methylation or histone deacetylase inhibition resulting in reduced or lost expression [16, 23], single nucleotide polymorphisms (SNPs) in pri- and pre-miRs as well as in miR biogenesis pathway genes [24–26], loss or gain of chromosomal material since miR encoding genes are commonly located in fragile regions associated with oral SCC (OSCC) [16, 27–29], or deregulation of key genes involved in miR biogenesis [30].

Despite the transformation rate of potentially malignant oral lesions to OSCC (31.4%) [31], clinical and histological characteristics have limited potential as predictors of transformation and do not aid in early diagnosis of HNSCC [32]. It has been shown that as many as 50% of HNSCCs may arise from apparently clinically normal mucosa, thus posing an inherent diagnostic challenge [33]. Although it is established that potentially malignant oral lesions (with the most common being leukoplakia and erythroplakia) and epithelial dysplasia are statistically more likely to progress to cancer, the actual mechanisms are poorly understood, and it is not inevitable that a dysplastic lesion will progress to cancer [33]. Thus upon clinical diagnosis of HNSCC, disease staging is often advanced, and prognosis is poor [34, 35]. The histopathological interpretation of tissue biopsies can be subjective and is thus prone to a considerable range of interpretation [35]. Similarly, no definitive, validated criteria exist for predicting which dysplastic lesions are most likely to progress to cancer over time [33, 36]. Given the current state of scientific knowledge, the presence of dysplasia can only be used to indicate that an oral lesion may have an increased risk of malignant transformation [37]. In spite of the considerable advances in diagnostic tools and treatment over the past three decades, mortality of HNSCC patients continues to be high, and current treatment modalities are still associated with many adverse effects and decreased quality of life [38].

## 2. Utilizing miRs in Early Diagnosis of HNSCC

Overexpression of oncomirs in tumours contributes to oncogenesis by inhibiting tumour suppressor genes and/or genes that control cell differentiation or apoptosis. For example, the *p53* gene is inhibited by miR-372/373. Other known oncomirs are miR-17-92, miR-21, and miR-155 [3, 19, 32, 39]. Some miRs have oncogenic properties on deregulation [15, 40]. For example, upregulation of mir-21 leads to inhibition of apoptosis [41], while its downregulation leads to chemoresistance [42]. Conversely, miRs may be characterized as tumour suppressors if their normal action opposes oncogenesis (e.g., let-7, and miR-15a/16-1, miR-34a, miR-143/145) [19, 43]. 

miRs are tissue specific and bind to the 3′ untranslated (3′-UTR) region of their target mRNA(s), resulting in a profound control over gene expression at the posttranscriptional level [6, 13, 14, 19, 44]. Significantly, some miRs may be oncogenic in one cell or tissue type but tumour-suppressive in another, depending on the tissue context and target genes [19]. Consequently specific miR patterns may be used to distinguish cancer cells from normal cells, as well as to identify the tissue of origin in carcinomas with unknown primary tumour, forming a means to early diagnosis of HNSCC [14, 43, 45, 46]. It has been suggested that specific miRs can be used as diagnostic markers for HNSCC diagnosis, such as overexpression of miR-21 and -205 [15, 47]. Avissar et al. [48] have claimed a sensitivity of 92% and specificity of 93% in classifying HNSCC using the expression ratio of miR-221 to miR-375. Furthermore, miRs can also discriminate between subtypes of a specific type of cancer and precise oncogenic abnormalities [16].

**Figure 1:**
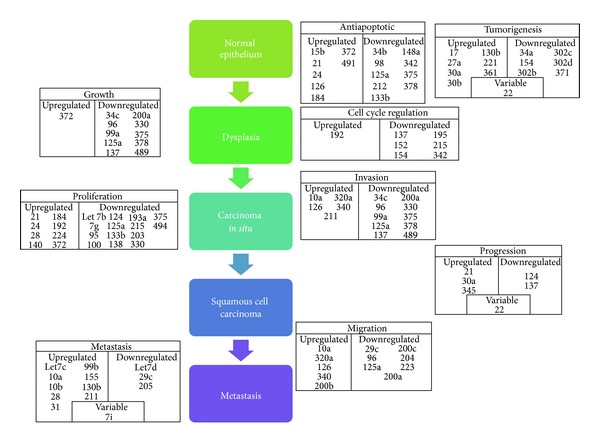
The role of miRs in regulating the transformation of normal squamous epithelial cells into carcinoma cells, ultimately resulting in metastasis (adapted from [3]).

miRs may also be used as diagnostic indicators of metastatic disease [15]. The hallmarks of cancer progression have been described as self-sufficiency in growth signals, ignorance of antigrowth signals, apoptosis evasion, limitless replicative potential, sustained angiogenesis, and tissue invasion and metastasis [49].  Figure 1 shows the deregulated miRs in each stage of cancer progression, from normal epithelium to metastatic cancer [3]. Barker et al. [50] found that miR profiles were distinct and specific for HNSCC in the tonsil, base of tongue, and postnasal space. Furthermore, the authors established that miR expression profiles between primary cancer and its nodal metastatic disease were consistent with the implication that miR profiles may be used as a diagnostic tool to determine whether the nodal metastasis is from the oral cavity, particularly when the primary tumour cannot be identified—a significant advantage of miR profiling [15, 16, 50]. Conversely, Hui et al. [51] did not find distinct expression profiles between the three subsites investigated—the hypopharynx, oropharynx, and larynx. This conflicting data may be due to technical problems as well as differences in stage, grading, and sampling from multiple anatomical sites but signifies the need for further analysis [40].

miRs further have a prognostic importance in determining the survival of patients with HNSCC [15]. For example, it has been suggested that high expression of mir-21 can be used as an independent predictor of poor survival for patients with tonsillar SCC and a significantly lowered 5-year survival in patients with HNSCC [41, 52], while lowered miR-205 expression [53] and increased miR-451 expression [51] have been significantly correlated with locoregional relapse of HNSCC irrespective of disease severity at diagnosis and treatment.

miRs may also be used as biomarkers in bodily fluids such as blood and saliva, since they have been shown to circulate stably in both healthy and cancer patients [15, 16]. This stability is due to their inclusion in lipid or lipoprotein complexes, such as apoptotic bodies, microvesicles, or exosomes, which prevent their degradation by RNAses [3]. Amplified plasma levels of miR-184 (in tongue carcinoma) [54], -31 [55], and -24 [29] (both in oral carcinoma) have been detected when compared with case-controlled individuals. miR-125a and -200a are two salivary miRs that have been shown to be substantially reduced in oral carcinoma patients versus healthy controls [56]. Moreover, plasma miRs were shown to be reduced after tumour resection, implying that these miRs may be released from cancerous tissues into circulation and their potential use as a marker for disease progression [54, 55]. This was seen in plasma and salivary miR-31 [57] and plasma miR-184 [45]. Further research is required in this area but positive results may lead to noninvasive diagnostic tests that will enable surgeons to determine if the margins are clear on a molecular level, which may decrease metachronous recurrence rates. Circulating miRs therefore have the potential to be powerful, noninvasive HNSCC biomarkers [15].

## 3. miRs in the Transition from Dysplasia to HNSCC

Despite the increasing number of studies on miR expression in HNSCC, there remain few publications that have investigated the deregulation of miRs in the transition process from dysplasia to malignancy. 

In an investigation of miR precursors in oral leukoplakias (OL), Xiao et al. found an upregulation of both miR-31 and miR-31* [58]. miR-31* was negatively associated with recurrent/newly formed oral leukoplakias, and they hypothesized that miR-31* may play an important role during OL progression via the regulation of fibroblast growth factor 3 (FGF3) [58]. This was consistent with miR expression profile findings in the prospective translational study by Lajer et al. [40], who examined the global miR expression in a series of consecutive tumours or biopsies obtained from patients with OSCC and pharyngeal SCC. Of the one hundred and fourteen miRs differentially expressed between OSCC and normal epithelium, the upregulation of miR-31 and downregulation of miR-375 were found as the most significant aberrations [40]. There is thus evidence to suggest that the upregulation of miR-31 may be an early event in the transition process from dysplasia to OSCC; however, clear elucidation of its role in tumour progression and of its predictive value still requires further investigation. 

Clague et al. conducted a case-control study investigating the association between miR-related gene polymorphisms and risk of oral potentially malignant lesions (OPML) [59]. It was found that an increased risk of OPML was noted with increasing number of unfavourable genotypes, with patients with at least one variant allele of mir26a-1:rs7372209 having a significantly increased risk of OPML [59]. However, due to the retrospective nature of the study and lack of patient followup, they were unable to conclude the viability of these OPML risk alleles as potential markers for risk of progression to HNSCC [59].

In the only study to date investigating the miR expression profiles associated with progression of leukoplakia to oral carcinoma, Cervigne et al. [32] quantified miR expression changes in leukoplakia and same-site OSCC in 43 sequential progressive samples from 12 patients and four nonprogressive leukoplakias from four different patients. They succeeded in identifying an miR signature associated with progression, which was also validated using quantitative RT-PCR in an independent cohort of 52 progressive dysplasias and OSCCs and five nonprogressive dysplasias [32].

Cervigne et al. found that miR-21, miR-181b, and miR-345 were consistently increased in oral dysplasia and associated with lesion severity, with global miR expression profiles being able to distinguish progressive leukoplakia/OSCC from nonprogressive leukoplakias/normal tissues [32]. Overall, one hundred and nine miRs were highly expressed exclusively in progressive leukoplakia and invasive OSCC, with a multi-miR prognosis predictor built consisting of a set of eight miRs derived using a classical training-testing set of samples. They concluded that overexpression of miR-21, miR-181b, and miR-345 had highly significant associations with progressive leukoplakia lesions and as such could play a role in malignant transformation and may potentially be useful as an miR signature for identifying leukoplakias at risk of malignant transformation [32]. All three of these miRs were upregulated in the OSCC samples as profiled by Lajer et al. in 2011 [40]. miR-181b was also found to be more highly expressed in patients with lymph node metastases from oral cavity cancer; however, it was noted that the number of patients with lymph nodes metastasis included in the study was too low to allow for the formulation of a distinct signature, and the changes noted were moderate [40]. Nonetheless, these observations lend evidence to suggest that these miRs may have diagnostic as well as prognostic value.

## 4. miRs in HNSCC Surgical Margins

To date, there has only been one study investigating the role of miR in surgical margins. Santhi et al. analysed 72 miRs reported to be differentially expressed in OSCC and detected decreased expression of miR-125a, miR-184, and miR-16 and an increased expression of miR-96 in both progressive oral mucosal samples and dysplastic surgical margin samples [60]. Further studies are required to define a broader set of miR profiles within a wider range of surgical specimen samples and to correlate results with patient outcomes.

## 5. miR Deregulation in HNSCC Related to Specific Risk Factors

### 5.1. HPV-Negative HNSCC (Tobacco, Alcohol, and Areca Nut Use)

Oral and laryngeal carcinomas are the most common types of HNC in smokers, who are ten times more likely to experience cancer than nonsmokers, while pharyngolaryngeal carcinomas commonly develop with alcohol intake [61, 62]. The concurrent use of tobacco and alcohol has a synergistic (greater than multiplicative joint) effect on the risk of developing HNSCC [3, 6]. The expression of miR-155 circulating in plasma/serum has been associated with a greater risk of oesophageal carcinoma in tobacco and alcohol users [63]. Through multivariate analyses, Avissar et al. [52] found miR-375 expression levels to increase with alcohol ingestion, with higher expression in pharyngeal and laryngeal tumours. 

Overexpression of miR-23a has been linked to areca nut extract exposure [64]. It also targets Fanconi anaemia complementation G (FANCG), inhibiting its expression and thus promoting oncogenesis [64]. Hung et al. identified an upregulation of mir-146a associated with area nut extract exposure, which subsequently enhanced the oncogenicity of OSCC cells [65]. A recent study into miR gene polymorphisms and susceptibility to environmental factors leading to oral carcinoma found four miR gene polymorphisms which may have increased susceptibility to oral carcinoma associated with betel nut chewing and tobacco [66].

Studies have implicated cigarette smoke in miR deregulation [67, 68] with the high toxicity and mutagenicity of cigarette smoke which correlated in lung carcinomas with damage to miRs located at fragile sites of the genome [69, 70] and with deregulation of miR regulatory mechanisms such as the p53 pathway [71, 72]. A recent study using tiling low-density arrays identified widespread changes in the miR expression profile of oral fibroblasts exposed to cigarette smoke condensates, promoting a phenotype which increases oral cancer migration [73].

### 5.2. HPV-Positive HNSCC

There has been a recent rise in tongue and oropharyngeal carcinomas that are unrelated to the use of tobacco products, while the incidence of other HNSCCs has decreased in the United States [74–78]. The human papillomavirus (HPV) has been detected in 26.2% of dysplastic leukoplakia and other potentially malignant intraepithelial oral neoplasms [79], emerging as a major aetiological factor and creating a new and enlarging subset of HNSCC [74, 80].

Lajer et al. [14] identified a set of core miRs implicated in the known HPV pathogenesis in HPV + HNSCC, namely, miR-15a/miR-16/miR-195/miR-497 family, miR-143/miR-145, and the miR-106-363 cluster. Gao et al. [81] investigated the miR profile in HPV+ oropharyngeal SCC and found that five miRs were significantly correlated—miR-9, -223, -31, -18a, and -155. Wald et al. [82] studied the miR expression profile in HPV16+ and HPV-HNSCC cell lines. miR-363, -33, and -497 were upregulated, while miR-155, -181a, -181b, -29a, -218, -222, -221, and -142-5p were downregulated. In a recent study, Hui et al. [83] found upregulated miR-20b, -9, and -9* associated with HPV/p16 status in oropharyngeal carcinoma. They also identified three candidate prognostic miR sets significantly associated with overall survival (miR-107, -151, and -492), disease-free survival (miR-20b, -107, -151, -182, and -361), and distant metastasis (miR-151, -152, -324-5p, -361, and -492), independent of p16 status [83]. 

In summary, as cited in a recent review article, Let-7, miR-125a/b, miR-200a, miR-133a/b, and miR-100 are considered tumour-suppressive miRs in HNSCC, while miR-106b-25 cluster, miR-17-92 polycistron, and miR-106a are oncogenic miRs in HNSCC [5]. Expression of miR-245, -21, and -181b is increased in leukoplakias transforming to OSCC and invasive OSCC [5]. The ratio miR-221 : miR-375 may be significant in discerning malignant HNSCC from normal tissue [5]. miR-205 has been associated with lymph node metastases [5].

It is evident that the results of most of these studies are conflicting and inconsistent, and no clear pattern has emerged [40]. Reasons for this may be due to the use of cell lines in some studies and formalin fixed paraffin embedded (FFPE) tissue samples in others, with different methods used for pathological staging and grading [40]. Samples may be from varying anatomical sites [40]. Cell lines cannot recapitulate miR profiling of solid tumours since culture conditions and clonal selection may drastically alter miR expression [40] but are used since they are inexpensive, easy to manipulate, and easily available for research purposes [84]. Moreover, the ongoing discovery of new miRs compels continued profiling in search of appropriate diagnostic and prognostic biomarkers [40].

There are large changes in miR expression compared to mRNA expression between normal cells and cancer cells thus aiding detection of differences [85]. miRs are also less prone to degradation and modification in FFPE tissue samples thus expediently supplying significant retrospective information [85]. It has been found that alterations in single miR expression have only a modest impact on individual protein expression [86, 87]. Global miR screening is therefore considered to be more useful in order to study collective changes in miR expression, since it is more likely that miRs work cooperatively *in vivo* to physiologically regulate proteins [88]. In accordance with this, Lajer et al. [14] observed that deregulation of a single miR implicates the collective action of a cluster of miRs or a whole miR family, consequently influencing multiple proteins in a complex manner.

## 6. miRs and Epigenetics

Epigenetic modifications at the promoter regions of genes (generally DNA methylation and histone modifications) and miR regulation at 3′-UTRs have emerged as two major regulatory mechanisms in eukaryotes, both of which can suppress gene expression [10]. It is likely that these two systems may complement each other since miRs tend to target genes with a low DNA methylation level in their promoter regions [10]. miRs are regulated by epigenetic mechanisms, similar to protein-coding genes, and the overexpression or underexpression of specific miRs in specific tumour types is a result of epigenetic aberrations in these tumour cells [10, 89]. These mechanisms include DNA methylation of miRs and aberrant expression of specific epigenetic regulators such as histone deacetylases (HDACs) or polycomb repressor complexes (PRC1 or PRC2) [10]. Since it has been found that approximately half of miR genes are associated with CpG islands, altered DNA methylation is a likely mechanism of miR regulation [16]. For example, in oral carcinoma, miR-137 is regulated by hypermethylation of the epigenetic targets—Cdk6, E2F6, NcoA2, and Lsd-1 [10].

There is also evidence that miRs also have specific epigenetic functions suggesting a fine-tuned feedback system [10, 16, 89]. This is achieved by firstly controlling the expression of important epigenetic regulators such as DNA methyltransferases, HDACs, PRC1, and PRC2—such miRs are termed epi-miRs [10]. Secondly, miRs may also have direct epigenetic functions by recruiting specific protein complexes to the promoter regions of genomic DNA [10]. Endogenous miRs are also able to activate or repress promoters directly, thus inducing or repressing specific genes directly [10]. Thus there is strong interdependence between the two gene regulatory mechanisms, and they are not entirely separable in their cooperation to establish the gene expression profile in specific cells [10]. Disruptions to this intricate network lead to various diseases including cancer [10].

## 7. Salivary Diagnostics

Saliva is an attractive medium for HNSCC biomarker detection due to the noninvasive nature of collection, its status as a proximal biofluid in the context of HNSCC, and the multitude of biomarkers that may be used to detect neoplastic change [90, 91]. Although the concept of salivary diagnostics in cancer is appealing, there has been difficulty in realising this due to the complexity surrounding HNSCCs as well as constituents in saliva [92–94]. 

Previous research into saliva as a source for biomarkers focused on proteomics [92], which has proved to be difficult due to protein polymorphism in saliva, protein instability, and degradation in stored samples [92] and due to saliva substrate concentrations being about a thousandfold less than those in blood [95].

With the discovery of miRs and their importance in the regulation of cellular processes, there has been an increasing interest in the potential utility of salivary miRs in HNSCC diagnostics and prognosis. In addition, it was reported that miR concentrations were higher in saliva compared to other bodily fluids [96]. Weber et al. compared miR expression levels in 12 body fluids, including traditionally used fluids such as plasma, and concluded that saliva had the highest concentration of miRs [96]. However, there remains a lack of studies investigating salivary miR levels in HNSCC. Liu et al. first reported the heightened miR-31 levels in saliva and plasma of OSCC patients with a subsequent decrease in miR-31 levels after surgery [55]. It should be noted however that the study did not conduct a sensitivity analysis due to a small sample size (*n* = 43). A subsequent study conducted by the same group compared salivary miR-31 levels of OSCC against oral verrucous leukoplakia (OVL) patients, with findings of significant differences in OSCC with OVL and healthy subjects [57]. This is significant as OVL has been reported to have a relatively higher risk of turning neoplastic [97], and this further hints at a potential utility of salivary miR diagnostics in discriminating between neoplastic and benign lesions. Further investigations should be conducted to better define the discriminatory power of salivary miR levels. In addition, the study also found greater miR-31 levels in the saliva of OSCC patients compared to those in the plasma [57]. The study concluded that salivary miR-31 as a cancer biomarker had greater sensitivity than that using plasma [57]. Langevin et al. found that there were higher miR-137 methylation levels in salivary mouth rinses of HNSCC patients [98], with a follow-up study concluding that patients with higher miR-137 methylation levels were associated with lower survival rates [99]. Given the current low literature base, more research is required to better elucidate the role of salivary miRs in HNSCC diagnostics and prognosis. 

## 8. Methodologies Used in Salivary miR Assays

Salivary diagnostics is unique in that the miR numbers can be up to a thousandfold less than those found in fresh tumor samples [100], as such there is an increased importance placed on proper sample collection and assessment methods. Normalisation for quantitative real-time polymerase chain reaction (qRT-PCR) assays has emerged as a significant source of differences between research groups. Normalisation for variations in miR values and cDNA synthesis is essential [101] between the cancer and healthy groups, especially with salivary miR [100] in order to allow comparable findings across studies. Two widely used normalisers in the literature for HNSCC miR analysis are endogenous small-nucleolar RNAs (snRNAs) such as RNU44, RNU48, RNU43, and RNU6B [102] as well as endogenous miRs such as Let-7a, miR-16, and miR-191 [103]. snRNA expression has been shown by Gee et al. to be variable in HNSCC patients, where the use of snRNA as normalisers acted to bias miR expression values [102]. There is also accumulating evidence of snRNAs playing a role in cancer [104]. Although the use of endogenous miRs in normalisation has been qualified in breast [105] and colorectal [106] cancers, there are no studies in the literature investigating the use of these miRs in HNSCC. This is important, as there is evidence that there are measurable changes in Let-7a [45, 47, 107, 108], miR-16 [45, 47], and miR-191 [50] levels in the context of HNSCC from the literature. 

In order to prevent problems with tissue context, some authors have described using a mean expression ratio of miRs expressed as a normalizer [109]. This approach has been qualified [110] and is well accepted [100]. Another approach is the use of synthetic control miRs that are spiked in before analysing to normalise the sample [111]. This approach has the advantage of having a quantifiable reference point that will not change [111]. However, some issues may arise with ensuring a homogenous quantity between experiments [100]. In view of these, investigators will be well served in choosing the right normaliser in the context of tissue types in conducting qRT-PCR to ensure that comparable results across studies are obtained.

There are two main forms of salivary samples used in the literature—whole saliva and the cell-free, supernatant phase that is obtained from progressive centrifugation. There has been debate over which is the more informative sample type since Park et al. first reported a different miR profile in the two samples, with the conclusion that the supernatant phase of saliva was the more informative one [56]. However, Patel et al. disagreed, stating that there was a higher resolution in the miR profile from whole saliva [90]. Spielman et al. were in agreement with Park et al. after using next generation sequencing (NGS) techniques to profile the RNA transcriptome in both phases of saliva [112]. This profiling technique is generally accepted to be the gold standard due to its accuracy and the ability to discern novel miRs [100]. Indeed, this debate in salivary diagnostics is not new, with previous research on salivary mRNA diagnostics raising similar contradictions [113–115]. The evidence appears to show that the supernatant phase of saliva contains more relevant miRs. In addition, reports of miR-containing, extracellular exosomes that resist degradation have bolstered the argument for using the supernatant phase in assay studies. 

## 9. Exosomal miRs in Saliva

Cell-free, endogenous salivary miR has been found to degrade at a much slower rate compared to nonsalivary miR when exposed to salivary ribonucleases [56, 116]. Moreover, Patel et al. found that Let-7b levels remained fairly constant when spiked with a RNA stabilizing agent and stored in room temperature for over 3 days [90]. There are two main hypotheses in the literature explaining this phenomenon. The first is that the miRs are packaged in exosomes, enabling the miRs contained within to be protected against degradation by salivary ribonucleases [117].

Exosomes are cell-secreted vesicles of 30–100 nm derived from the fusion of multivesicular bodies to plasma membranes [118] and are contained in the supernatant of saliva [119]. Exosomes have been thought to allow intercellular communication [120] through horizontal transfer between cells in different anatomic sites [121], as well to protect miRs from thermal, pH, freeze-thaw cycles, and extracellular ribonuclease [122] and is thought by some authors as the major source of miRs in saliva and serum [119]. Arroyo et al. contest this [123] although it should be noted that the study investigated plasma miR. There are no studies in the literature investigating the concentration of miR outside of exosomes in the supernatant phase of saliva.

The second hypothesis is that these miRs form a protein complex with Argonaute 2, increasing its stability in plasma and serum [123]. Although one group has found that up to 90% of miRs in plasma and serum are found in these complexes instead of in exosomes [123], there are no studies in the literature that have investigated this hypothesis in saliva. 

## 10. Novel Therapeutic Targets: The Small and Powerful

In addition to having considerable diagnostic and prognostic value, miRs are potential therapeutic targets or therapeutic agents depending on the type of mRNA(s) they affect [14]. miRs provide an unparalleled opportunity to target multiple molecules, usually in the context of a network, making them efficient in regulating distinct biological cell processes [16]. Synthetically designed miR mimics (agomiRs) [124], miR inhibitors (such as anti-miR oligonucleotides; AMOs) [41], miR antagonists (“antagomiRs”) [125, 126], and “miR sponges” [127] are some innovative methods of modulating oncogenic or tumour-suppressive pathways for therapeutic purposes. 

Strategies to target miRs in cancer may be direct, involving the use of oligonucleotides or virus-based constructs to either inhibit oncomir expression or to upregulate tumour suppressor miR expression, or indirect, where drugs are used to modulate miR expression by targeting their transcription and processing [16]. However, a number of challenges prevail in the development of a specific and efficient drug delivery system for miR-based drugs [15, 16]. Therapeutic RNA must exit the circulatory system, transit the cell membrane, and avoid endosomal vesicles to gain entry into the cytoplasm and access the target site [16]. Furthermore, nonconjugated therapeutic RNA (7–20 kDa) is likely to be cleared by phagocytic immune cells or by the kidneys, which filter out molecules less than 50 kDa [16]. While there is limited information on miR-based therapeutic targets in HNSCC, the following is a brief description of known miR therapeutic agents and targets.

Mature miR levels may be reduced by exogenously delivering synthetic double-stranded hairpin by complexing with lipids or delivery proteins: introduction of miR-34a induced apoptosis of experimental lung metastasis of murine melanoma and suppressed cellular proliferation in two colon cancer cell lines and may also be effective in HNSCC cells [128, 129]. Unmodified dsRNAs are likely to be degraded by nucleases *in vivo*, limiting the use of this class of compound to privileged local environments where local administration is possible (e.g., intranasal delivery in mouse lung cancer model) [85]. For stable miR reintroduction, the expression can be enforced by a viral vector with Pol III promoters, which have the advantage of providing high expression of miRs from well-defined transcription start and termination sites [129–131]; however, they have no cell specificity [16]. In contrast, RNA Pol II promoters can express pri-miRs, allowing for tissue-specificity or induced ectopic miR expression [132]. The latter two methods may be effective in the reintroduction of downregulated miRs in HNSCC (e.g., miR-375 [40]).

However, there are many hazards of reintroducing an miR with a viral system [16]. The site of integration of the delivered material to host DNA is unpredictable, with the associated risks of insertional mutagenesis and activation of protooncogenes [16]. The use of retroviral vectors (which, along with lentiviral vectors, integrate their DNA into the host genome) is limited to actively dividing cells, while adenoviral vectors (which remain unintegrated into the host genome, replicating as an autonomous unit) tend to induce a strong immunological response [16, 133].

AntagomiRs are a class of chemically engineered oligonucleotides able to silence endogenous miRs [16, 126]. AntagomiRs of upregulated miRs in HNSCC would revert to the effects of upregulation; for example, delivery of antagomir-155 into KB cells overexpressing miR-155 in nude mice decreased cell viability and increased apoptosis [125]. The interfering nanoparticle (iNOP) is a novel therapeutic agent which may be complexed with antagomiRs and delivered intravenously, enhancing the delivery of the antagomiR [134]. The effects of iNOP-stabilised anti-miR-122, which silenced miR-122 in mice, were found to be long lasting and did not induce an immune response [134].

miR loss of function may be achieved by AMOs (chemically modified anti-miR oligonucleotides), which have high specificity and binding affinity to RNA but lack an effective delivery mechanism into target tissues [41, 128]. Inhibition of mir-21 with AMO in tongue SCC cell lines has been shown to induce apoptosis and reduce survival and anchorage-independent growth [41]. Furthermore, repeated injection of miR-21 AMO suppressed tumour formation in nude mice reducing cell proliferation and inducing apoptosis [41].

Oncomirs in HNSCC may be inhibited using miR sponges or miR-masks. miR sponges are miR inhibitory transgenes expressing an mRNA containing multiple tandem binding sites for an endogenous miR (with the potential to block entire miR families), thus able to stably interact with the corresponding miR and prevent the association with its endogenous targets [16, 127]. In contrast, miR-masking antisense oligonucleotides technology consists of fully complementary antisense oligonucleotides to complementary miR binding sites in the 3′-UTR of a specific target mRNA, thus disrupting the interaction between specific miR-mRNA pairs [16, 131]. While unwanted effects or off-target effects are greatly reduced in this method, this might be a disadvantage in cancer therapy where targeting multiple pathways might be desirable [16].

In addition to targeted therapies and chemotherapies, miRs could also alter sensitivity to radiotherapy [132], suggesting that the activation or suppression of implicated miRs would enhance the outcome of radiotherapy. Furthermore epigenetic drugs (e.g., DNA demethylating agents and histone deacetylase inhibitors) are capable of reversing irregular methylation or acetylation [16]. The action of such drugs could restore tumour-suppressive miR expression and revert a tumoral phenotype [16].

The majority of the aforementioned methods are in the experimental phase, but development of animal models to study cancer-associated miRs and improvement of miR/anti-miR delivery efficiency *in vivo* are fundamental to translating these research advances to medical practice [16]. Furthermore, most of these strategies target a single miR or a family of miRs [16]. Since multiple miRs coordinate in cancer pathogenesis with multiple miR-transcriptome interactions, future strategies should aim to reprogramme aberrant miR networks in cancer [16]. This may be achieved by targeting components of miR biogenesis machinery or elements of regulatory networks (such as the epigenetic programme) [16, 133]. Furthermore, it has been shown that miRs are actively reexpressed after treatment with these drugs, contributing to their therapeutic benefits [16]. Nevertheless, the full potential of these drugs remains to be verified. 

## 11. Conclusion

Current research has identified deregulated miRs in HNSCC oral premalignant lesions; however, a wide range of miRs have been implicated, regulated by cellular and epigenetic factors. miR signatures with diagnostic accuracy in tumour tissue samples, premalignant lesion samples, and saliva samples are yet to be validated in large clinical trials. This would lead to the development of novel therapeutic targets and agents with the overarching aim of providing customised patient-centred management leading to improved prognosis for HNSCC patients.
